# Immunophenotyping of the PD-L1-positive cells in angioimmunoblastic T cell lymphoma and Hodgkin disease

**DOI:** 10.1186/s13104-020-04975-w

**Published:** 2020-03-07

**Authors:** Markus Tiemann, Vera Samoilova, Dmitri Atiakshin, Igor Buchwalow

**Affiliations:** 1 Institute for Hematopathology, Hamburg, Germany; 2 Research Institute of Experimental Biology and Medicine, Burdenko Voronezh State Medical University, Voronezh, Russia

**Keywords:** Angioimmunoblastic T-cell lymphoma, Hodgkin lymphoma, Receptor PD-1, Ligand PD-L1

## Abstract

**Objective:**

Programmed death-1 (PD-1) and its ligand PD-L1 are now used as predictive biomarkers to guide clinical decisions. Precise characterization of PD-L1-positive cells may contribute to our knowledge of which patients derive benefit from the PD-L1 blockade therapy.

**Results:**

To address this issue, we performed immunophenotyping of PD-L1-positive cells in Hodgkin lymphoma and in angioimmunoblastic T cell lymphoma (AITL) employing multiple immunofluorescent immunolabeling. We found that PD-L1-positive cells and PD-1-positive cells both in Hodgkin lymphoma and in AITL belong to two completely different cell lineages. In both lymphomas, PD-1 was found exclusively in T-lymphocytes, whereas PD-L1 was revealed in the tumor microenvironment cells including macrophages. PD-L1 was also detected in CD30-positive cells in Hodgkin lymphoma but not in AITL. The marker of B-cell lineage, CD20, was not detectable in PD-L1-positive cells both in AITL and in Hodgkin. Our study highlights the importance of comprehensive assessment of PD-1/PD-L1 regulatory pathways for employing PD-L1 as a predictive biomarker in clinical practice. PD-L1-antibody therapy is proven in Hodgkin lymphoma. Comparative immunophenotyping of the PD-1/PD-L1 axis provides a support for attempts to prove this principle also for AITL.

## Introduction

In 1992 the team of Tasuku Honjo in a screen for genes, involved in apoptosis, discovered a protein expressed on the surface of a subset of immune cells known as T cells and named this protein PD-1 (short for Programmed death-1) [1]. PD-1 is expressed predominantly on activated T cells [2]. In 1999, a ubiquitous antiapoptotic receptor on cancer cells was reported from the Mayo Clinic [3]. Originally it was named B7-H1 but later renamed PD-L1, because it was identified as a ligand of PD-1. The binding of PD-1 to its ligand PD-L1 induces apoptosis or exhaustion in activated T cells thus preventing the immune system from killing cancer cells [4, 5]. PD-L1 is not present in large quantity in normal tissue, but it is upregulated in a variety of tumors [6, 7]. Upregulation of PD-L1 allows cancers to evade the host immune system [8, 9]. Development of therapeutic anti-PD-1/PD-L1 monoclonal antibodies leading to the reactivation of specific antitumor immune response has emerged as a promising strategy for hematological malignancy therapy including various lymphoma arts [10–16]. PD-1/PD-L1 immune-checkpoint blockade therapies reactivate the specific antitumor immune response [17–22]. However, to predict patients who are likely to respond to treatment with PD-1/PD-L1 blockers still remains a challenge [23].

Precise characterization of PD-L1-positive cells may contribute to our knowledge of which patients derive benefit from the PD-L1 blockade therapy [24, 25]. This study was aimed at the immunophenotyping of the PD-1/PD-L1 axis in Hodgkin lymphoma and in AITL. For immunofluorescent multiple immunolabeling we used antibodies to PD-L1, PD-1 and a panel of CD antibodies raised against diverse cell types.

## Main text

### Methods

#### Patients

15 AITL and 8 Hodgkin patients were included in this study. Informed consent was obtained from all subjects. The samples were retrieved from the files of the Institute for Hematopathology, Hamburg, Germany. Histological diagnoses were established according to the WHO classification [26, 27]. This study was conducted in accordance with the “Ethical Principles for Medical Research Involving Human Subjects” and approved by the Institutional Review Board of the Institute for Hematopathology, Hamburg, Germany.

#### Tissue probe stainings

Tissue probes were fixed in buffered 4% formaldehyde and routinely embedded in paraffin. Deparaffinized and rehydrated sections (1 µm thick) were subjected to antigen retrieval by heating in a steamer with sodium citrate buffer, pH 6.0, at 95 °C × 30 min. Blocking the endogenous Fc receptors prior to incubation with primary antibodies was omitted [28]. After antigen retrieval, sections were immunoreacted with primary antibodies (Additional file 1: Table S1). Bound primary antibodies were visualized using secondary antibodies listed in the Additional file 2: Table S2. Principally, immunohistochemical staining was performed according to the standard protocols described earlier [29–31].

#### Controls

Control incubations were: omission of primary antibodies or substitution of primary antibodies by the same IgG species (Dianova, Hamburg, Germany) at the same final concentration as the primary antibodies. The exclusion of either the primary or the secondary antibody from the immunohistochemical reaction, substitution of primary antibodies with the corresponding IgG at the same final concentration resulted in lack of immunostaining.

### Results

**Fig. 1:**
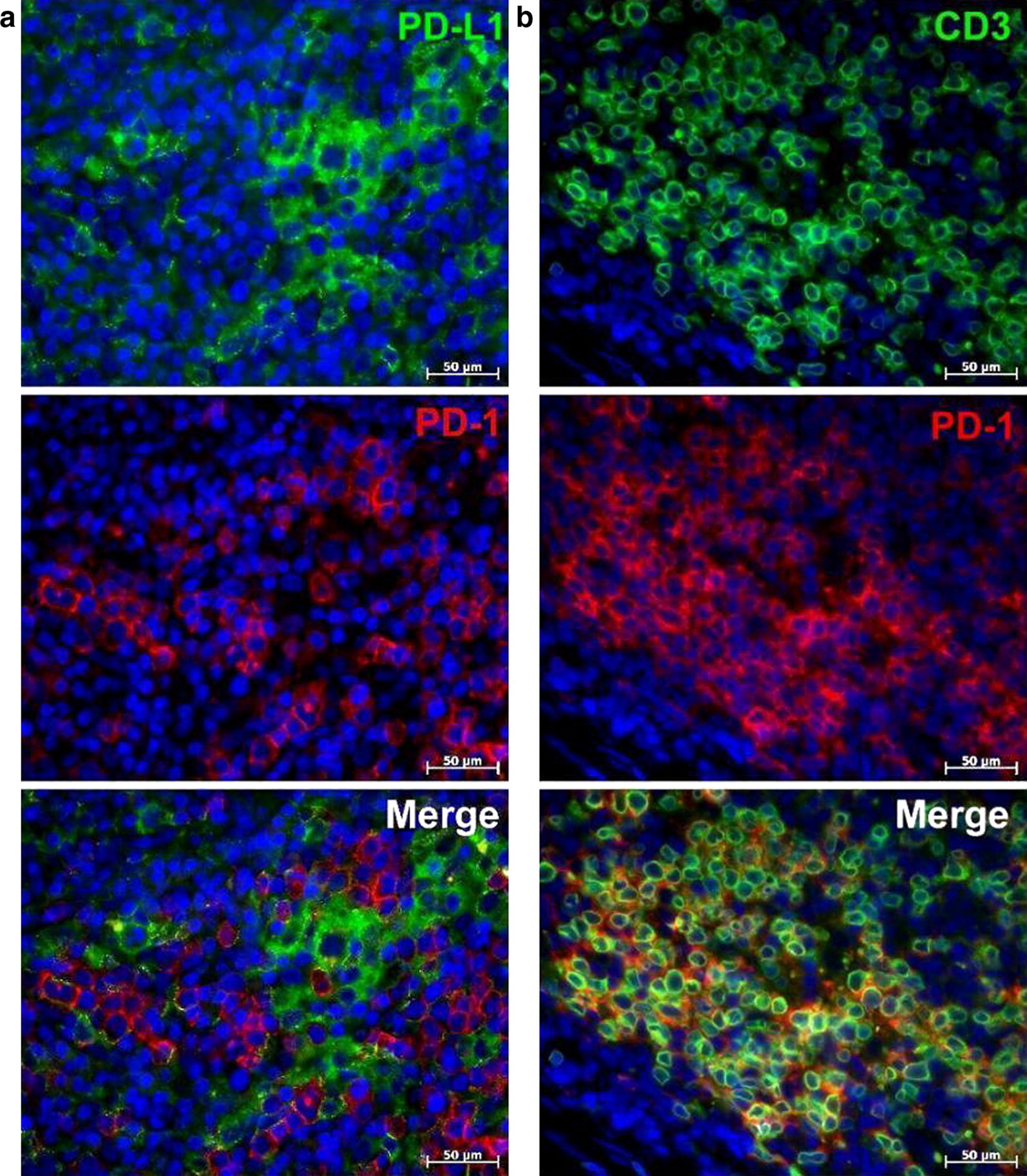
Immunofluorescent staining of PD-1, PD-L1 and CD3 in AITL. **a** Immunofluorescent double staining of PD-1 (Cy3, red) and PD-L1 (Alexa Fluor-488, green). PD-1 and PD-L1 are expressed poles apart from each other. **b** Immunofluorescent double staining of PD-1 (Cy3, red) and CD3 (Alexa Fluor-488, green). The majority of CD3-positive cells bear the PD-1 marker. Nuclei counterstained with DAPI (blue channel)

**Fig. 2:**
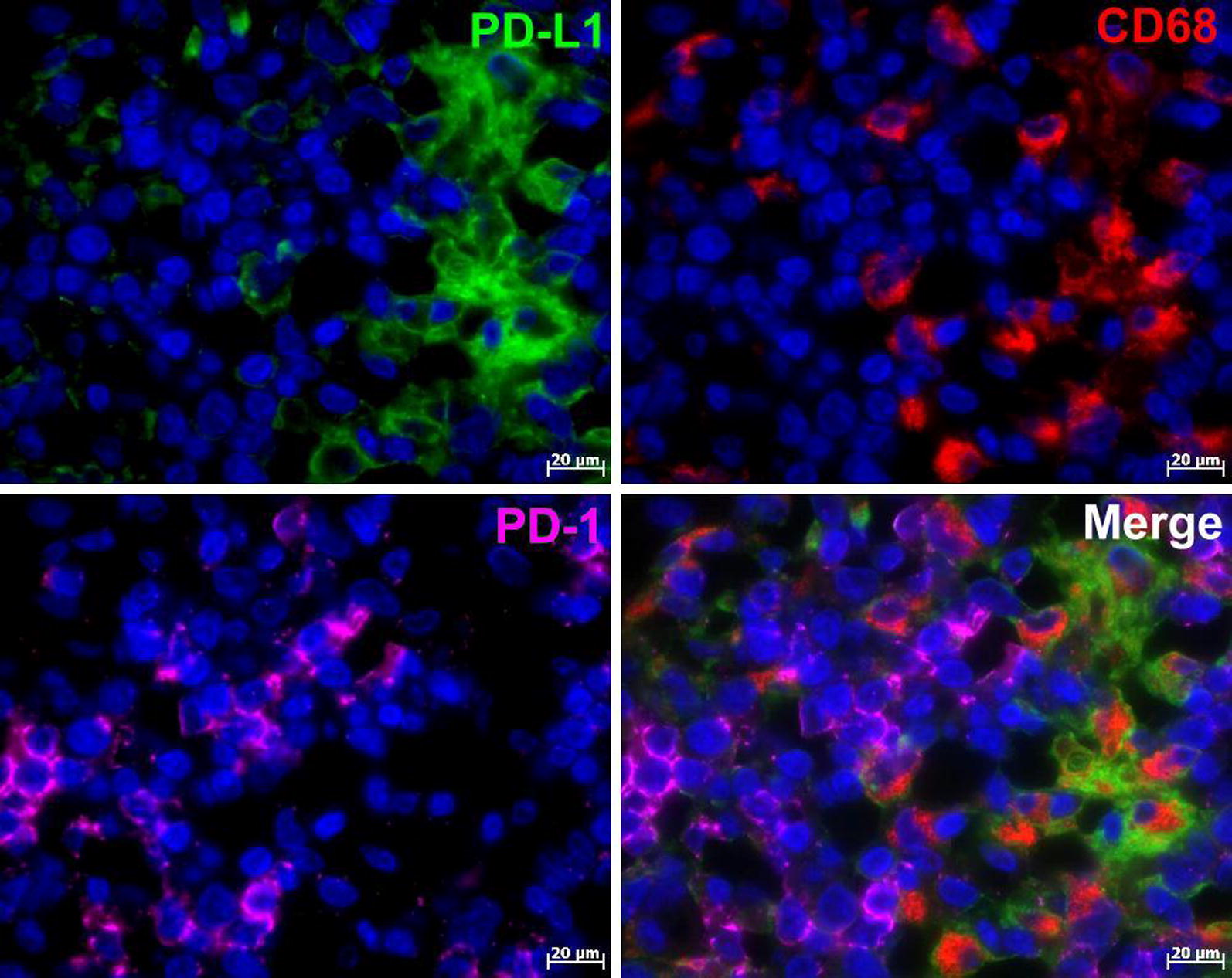
Immunofluorescent triple staining of PD-1, PD-L1 and CD68 in AITL: PD-L1 (Alexa Fluor-488, green), PD-1 (Alexa Fluor-647, magenta) and CD68 (Cy3, red). Nuclei counterstained with DAPI (blue channel). PD-1 and PD-L1 are expressed poles apart from each other, and only PD-L1- positive cells co-express CD68, the marker for macrophage lineage

Employing multiple immunofluorescent labeling of cellular components in tissue sections of the human AITL and Hodgkin lymphoma, we found that PD-1 and PD-L1 are localized in different cells (Fig. 1a; Additional file 3: Fig. S1). CD3, marker of T-cell lineage, was co-localized with PD-1 (Fig. 1b) and never with PD-L1. Triple immunofluorescent labelling for PD-1, PD-L1 and CD68 in tissues both of Hodgkin lymphoma and AITL (Fig. 2) revealed that CD68, marker for macrophage lineage, including monocytes and histiocytes, was solely and exclusively expressed in PD-L1+ cells and never in PD-1+ cells. This indicates that PD-1+ and PD-L1+ cells belong to completely different cell lineages.

For simultaneously detecting antigens from the same host species as presented in Fig. 2 and in Additional file 4: Fig. S2, we performed tyramide signal amplification (TSA) with the subsequent heat elution treatment after each immunostaining step [32, 33].

**Fig. 3:**
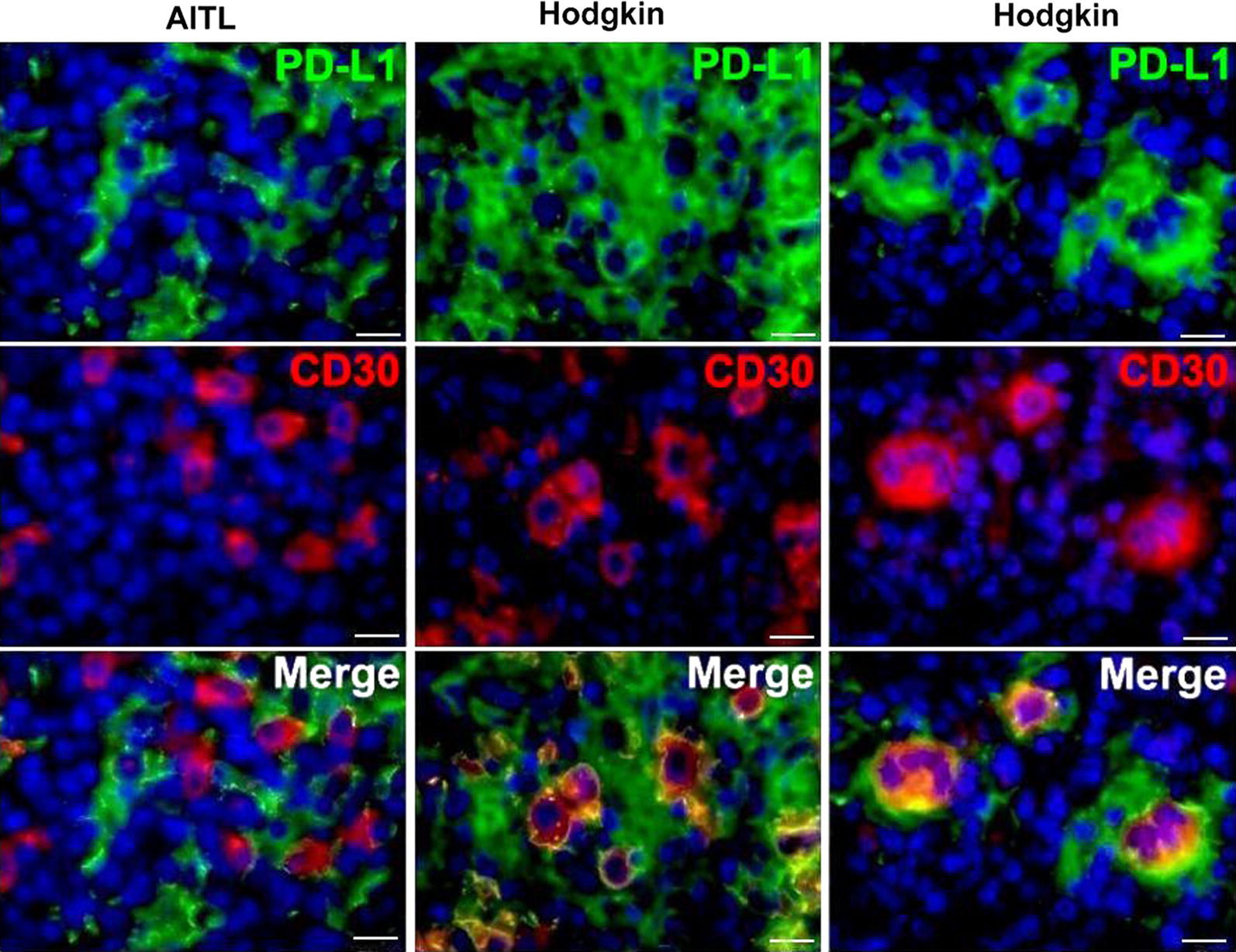
Immunofluorescent double staining of PD-L1 and CD30 in AITL and Hodgkin lymphoma: PD-L1 (Alexa Fluor-488, green), CD30 (Cy3, red). Nuclei counterstained with DAPI (blue channel). Unlike AITL, where CD30-positive cells no not co-express PD-L1, CD30+ cells in Hodgkin lymphoma bear PD-L1 antigen on the cell membrane. Scale bar 20 μm

CD30 expression in AITL and Hodgkin lymphoma is currently of great interest, because therapy targeting CD30 is of clinical benefit [34]. CD30, belonging to the tumor necrosis factor receptor superfamily, is highly expressed on Reed/Sternberg cells (RSC) and believed to be involved in tumorigenesis and tumor progression [35]. In this study, we found CD30-positive cells both in Hodgkin lymphoma and in most cases in the AITL. However, histotopographic relations of CD30 antigen with PD-1 and PD-L1 revealed some differences. As seen in Fig. 3, CD30-positive cells in AITL do not express PD-L1, whereas PD-L1 antigen in Hodgkin lymphoma was detected in CD30-positive cells including Reed–Sternberg cells (RSC), preferably at the periphery, possibly at the cell membrane. In contrast to PD-L1, PD-1 was not found in RSC (Additional file 5: Fig. S3). Our results confirm and extend prior studies of PD-L1 expression in RSC in Hodgkin lymphoma [6, 36].

It was earlier reported that CD30-positive cells exhibit a B-cell or a T-cell phenotype [37] and that RSC can derive from B lymphocytes [38, 39]. However in our study, we found that PD-L1-positive cells including rare malignant Hodgkin and RSC cells do not express CD20 (the marker of B-cell lineage) neither in Hodgkin lymphoma (Additional file 4: Fig. S2) nor in AITL (Additional file 6: Fig. S4). To prove a possible co-expression of CD20 in PD-L1+ cells in other tumors differing from AITL and Hodgkin lymphoma, we analyzed the probes of mediastinal lymphoma [4] taken as a control and in this case observed a definite co-localization of PD-L1 and CD20 (Additional file 7: Fig. S5).

Since it was assumed that CD10 antibody might be useful in the diagnosis of AITL [40], we decided to perform immunofluorescent double staining of PD-L1 and PD-1 vs CD10 in this lymphoma. We found that PD-L1 and CD10 are never co-localized in the same cells (Additional file 8: Fig. S6 a–c), whereas the majority of PD-1-positive cells revealed a co-expression of CD10 (Additional file 8: Fig. S6 d–f).

### Discussion

PD-L1 was originally characterized as a ubiquitous antiapoptotic receptor on cancer cells [3] and it has been proposed as potential target in cancer immunotherapy in human clinic [24, 25]. However, we found that cancer cells (CD10+ and CD30+ cells) in AITL lacked the expression of PD-L1 (Additional file 5: Fig. S3 and Additional file 8: Fig. S6), whereas CD30+ cells including RSC in Hodgkin lymphoma revealed a co-expression of PD-L1 on the surface of these cells (Fig. 3). This is consistent with reports that PD-L1 expression in malignant cells of various cancers varies from 0 to 50% [7]. Therefore PD-L1 expression by tumor cells cannot serve as an absolute biomarker of clinical response to checkpoint blockade in immunotherapy, while patients, by which malignant cell in the tumor lack PD-L1 expression, also responded positively to PD-L1 checkpoint blockade therapies [7, 21, 41].

Patients with overexpressed PD-L1 in the tumor microenvironment, have improved clinical outcomes with anti-PD-L1-directed therapy [24]. Therefore PD-L1 expression in the tumor microenvironment can be regarded as a more valuable biomarker to guide clinical decisions. In our study, PD-L1 was found in the tumor microenvironment richly expressed in cells of macrophage lineage (Fig. 2). Apparently antigen-presenting cells such as macrophages may serve as a main target in PD-L1 checkpoint blockade therapies in AITL and Hodgkin lymphoma.

Our findings that CD10 in AITL is co-localized with PD-1-positive T-cells but not with PD-L1-positive cells in the tumor microenvironment support the recent studies describing CD10 as a phenotypic marker that specifically identifies the tumor cells in 90% of AITL, including the early cases [42]. The presence of CD10-positive T-cells distinguishes AITL from other unspecified peripheral T-cell lymphomas, where no CD10-positive T cells are present. Some authors reported the utility of CD10 antibody as a diagnostic marker of AITL [40]. However, further studies correlating with the clinical course will be of interest in determining the biological significance of CD10 in the AITL.

In our study, we found that PD-L1-positive cells including RSC in Hodgkin lymphoma (Additional file 4: Fig. S2) and in AITL (Additional file 6: Fig. S4) do not express CD20 (the marker of B-cell lineage). It is in accord with reports that HRS cells are CD20 negative due to downregulation of the B-cell program and it is not surprising that they CD20 and PD-L1 do not co-localize together [4, 43]. Also, these cells universally express PD-L1 in most instances with amplifications of the PD-L1 locus. This issue must further be assessed in view of the reports on elevated PD-L1 expression on B cells in other tumors [44].

Hodgkin lymphoma and AITL are malignancies in which rare malignant cells are surrounded by an extensive but ineffective inflammatory/immune cell infiltrate including PD-1-positive T-cells and PD-L1-positive antigen presenting cells. This striking feature suggests that malignant cells in these lymphomas escape immunosurveillance and interact with immune cells in the cancer microenvironment for survival and growth. Enhanced PD-1/PD-L1 signaling in Hodgkin lymphoma [4] and likewise in AITL can make these both tumors uniquely sensitive to PD-1/PD-L1 blockade.

Some authors reported on the co-expression of PD-1/PD-L1 in tumors, but virtually they presented only a simultaneous expression of PD-1 and PD-L1 in a tumor tissue taken en bloc and not in the same cells [45]. We employed multiple immunofluorescent labeling of cellular components in tissue sections of the human AITL and Hodgkin lymphoma and found that PD-1 and PD-L1 are localized in different cells, which implies that PD-1+ and PD-L1+ cells both in AITL and in Hodgkin lymphoma belong to two different cell lineages.

To summarize, our data allowed us to draw several conclusions. PD-1+ and PD-L1+ cells in the AITL and Hodgkin lymphoma are never co-localized in the same cells and therefore belong to two different cell lineages. Generally, the immunophenotype of PD-1+ and PD-L1+ cells in AITL and Hodgkin is similar, with an only exception relating to CD30 and CD10. In both lymphomas, PD-1 was found exclusively in T-lymphocytes, whereas PD-L1 was revealed in antigen-presenting cells—macrophages. PD-L1 was also detected in CD30-positive cells in Hodgkin lymphoma but not in AITL. The marker of B-cell lineage, CD20, was not detectable in PD-L1-positive cells both in AITL and in Hodgkin. HRS cells are CD20 negative due to downregulation of the B-cell program and it is not surprising that they CD20 and PD-L1 do not co-localize together. Characterization of PD-L1+ cells in these lymphomas may contribute to the development of effective approaches to the delivery anti-PD-L1 antibodies to tumors in PD-L1 blockade therapy for patients suffering from PD-L1-expressing tumors. PD-L1-antibody therapy is already proven in Hodgkin lymphoma, but there is no information about PD-L1-antibody effect in AITL. Our findings may provide further insight into an opportunity of at least an experimental attempt or clinical study as proof of the similar approach to improve the results of treatment in AITL.

## Limitations

It would be interesting to what proportion of CD30+ cells in AITL are CD3+ . This question was beyond the scope of our study but it must further be assessed since that has therapeutic implications in this disease.

## Supplementary information

**Additional file 1: Table S1.** Primary antibodies used in this study.

**Additional file 2: Table S2.** Secondary antibodies and other reagents.

**Additional file 3: Figure S1.** Immunofluorescent double staining of PD-1 and PD-L1 in Hodgkin lymphoma: PD-L1 (Alexa Fluor-488, green), PD-1 (Cy3, red). Nuclei counterstained with DAPI (blue channel). PD-1 and PD-L1 are expressed poles apart from each other.

**Additional file 4: Figure S2.** Immunofluorescent triple staining of PD-1, PD-L1 and CD20 in Hodgkin lymphoma: PD-1 (Alexa Fluor-488, green), PD-L1 (Alexa Fluor-647, magenta) and CD20 (Cy3, red). Nuclei counterstained with DAPI (blue channel). CD20 was not found in Reed/Sternberg cells (RSC).

**Additional file 5: Figure S3.** Double immunostaining of PD-1 and CD30 in Hodgkin lymphoma: (**a**, **b**) Immunofluorescent double staining of PD-1 (Alexa Fluor-488, green) and CD30 (Cy3, red). RSC intensely express CD30 in the cytoplasm and do not bear PD-1 antigen. (**c**) Double immunoenzyme staining for CD30 (red) and PD-1 (brown). Nuclei counterstained with hematoxylin. RSC do not bear PD-1 antigen.

**Additional file 6: Figure S4.** Immunofluorescent double staining of PD-L1 and CD20 in AITL: PD-L1 (Alexa Fluor-488, green), CD20 (Cy3, red). Nuclei counterstained with DAPI (blue channel). PD-L1+ cells do not co-express CD20.

**Additional file 7: Figure S5.** Immunofluorescent double staining of PD-L1 and CD20 in mediastinal lymphoma: PD-L1 (Alexa Fluor-488, green), CD20 (Cy3, red). Nuclei counterstained with DAPI (blue channel). Co-expression of PD-L1 and CD20 in the same cells in mediastinal lymphoma is manifested by a hybrid orange colour.

**Additional file 8: Figure S6.** Immunofluorescent double staining of PD-L1 (FITC, green) and PD-1 (Alexa Fluor-488, green) vs CD10 (Cy3, red) in AITL. Nuclei counterstained with DAPI (blue channel). Whereas PD-L1 and CD10 were never found in the same cells (**a**–**c**), the majority of PD-1-positive cells revealed a co-expression of CD10 manifested by a hybrid orange coulour (**d**–**f**).

## Data Availability

All data and materials are available on reasonable request. Address to I.B. (email: buchwalow@pathologie-hh.de) or M.T. (email: mtiemann@hp-hamburg.de) Institute for Hematopathology, Hamburg, Germany.

## Abbreviations

PD-1: Programmed death-1 and its ligand PD-L1
AITL: Angioimmunoblastic T-cell lymphoma
RSC: Reed–Sternberg cells
