# Supplementary material for: Immunophenotyping of the PD-L1-positive cells in angioimmunoblastic T cell lymphoma and Hodgkin disease
Source: BMC Res Notes. 2020 Mar 7;13:139. doi: 10.1186/s13104-020-04975-w (PMC7060537; doi:10.1186/s13104-020-04975-w)
Supplement: Supplementary file 1 — Additional file 1: Table S1. Primary antibodies used in this study. [file 13104_2020_4975_MOESM1_ESM.docx]

**Table S1. Primary antibodies used in this study**

| **Antibodies/Clone** | **Host** | **Source** |
| --- | --- | --- |
| CD3 (PS1) | mouse monoclonal Ab | AbCam, United Kingdom |
| CD3 (2GV6) | rabbit monoclonal Ab | Ventana/Roche, Germany |
| CD8 (SP57) | rabbit monoclonal Ab | Ventana/Roche, Germany |
| CD10 (56C6) | mouse monoclonal Ab | Novocastra/Leica, United Kingdom |
| CD20 (L26) | mouse monoclonal Ab | Ventana/Roche, Germany |
| CD30 (Ber-H2) | mouse monoclonal Ab | Ventana/Roche, Germany |
| CD68 (KP-1) | mouse monoclonal Ab | Ventana/Roche, Germany |
| PD-1 (NAT105) | mouse monoclonal Ab | Cell Marque, USA |
| PD-L1 (ZR3) | rabbit monoclonal Ab | Zeta Corporation, USA |
