# Supplementary material for: Immunophenotyping of the PD-L1-positive cells in angioimmunoblastic T cell lymphoma and Hodgkin disease
Source: BMC Res Notes. 2020 Mar 7;13:139. doi: 10.1186/s13104-020-04975-w (PMC7060537; doi:10.1186/s13104-020-04975-w)
Supplement: Supplementary file 2 — Additional file 2: Table S2. Secondary antibodies and other reagents. [file 13104_2020_4975_MOESM2_ESM.doc]

**Table S2. Secondary antibodies and other reagents**

| **Antibodies** **and other reagents** | **Source** | **Dilution** | **Label** |
| --- | --- | --- | --- |
| Goat anti-mouse IgG Ab (#A21236) | Invitrogen,  Darmstadt, Germany | 1/100 | Alexa Fluor 647 |
| Goat anti-rabbit IgG Ab (#A21245): | Invitrogen,  Darmstadt, Germany | 1/100 | Alexa Fluor 647 |
| Goat anti-mouse IgG Ab (#115-165-166) | DIANOVA  Hamburg, Germany | 1/200 | Cy3 |
| Goat anti-rabbit IgG Ab (#A-11034) | DIANOVA  Hamburg, Germany | 1/200 | Cy3 |
| Goat anti-mouse IgG A (#A-11029 | Invitrogen  Darmstadt, Germany | 1/200 | Alexa Fluor 488 |
| Goat anti-rabbit IgG Ab (#A-11034) | Invitrogen  Darmstadt, Germany | 1/200 | Alexa Fluor 488 |
| AmpliStain™ anti-Mouse 1-Step HRP (#AS-M1-HRP) | SDT GmbH,  Baesweiler, Germany | ready-to-use | HRP |
| AmpliStain™ anti-Rabbit 1-Step HRP (#AS-R1-HRP) | SDT GmbH,  Baesweiler, Germany | ready-to-use | HRP |
| 4',6-diamidino-2-phenylindole (DAPI, #D9542-5MG) | Sigma,  Hamburg, Germany | 5 µg/ml | w/o |
| VECTASHIELD® Mounting Medium (#H-1000) | Vector Laboratories, Burlingame, CA, USA | ready-to-use | w/o |
| CC2 solution (# 950-223) | Ventana | ready-to-use | w/o |
| TSA Plus Fluorescein (#NEL741E001K) | PerkinElmer,  Rodgau Germany | 1/100 | Fluorescein |
| TSA Plus Cyanine 3 (#NEL744E001KT) | PerkinElmer,  Rodgau Germany | 1/100 | Cyanine 3 |
