# Supplementary figures and images for: Immunophenotyping of the PD-L1-positive cells in angioimmunoblastic T cell lymphoma and Hodgkin disease
Source: BMC Res Notes. 2020 Mar 7;13:139. doi: 10.1186/s13104-020-04975-w (PMC7060537; doi:10.1186/s13104-020-04975-w)

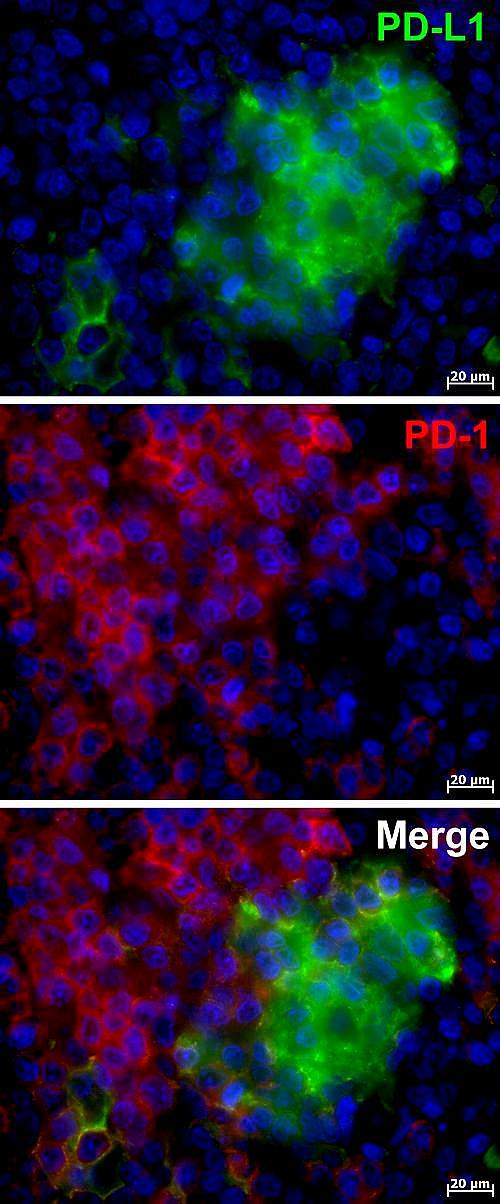

Supplement: Supplementary file 3 — Additional file 3: Figure S1. Immunofluorescent double staining of PD-1 and PD-L1 in Hodgkin lymphoma: PD-L1 (Alexa Fluor-488, green), PD-1 (Cy3, red). Nuclei counterstained with DAPI (blue channel). PD-1 and PD-L1 are expressed poles apart from each other. [file 13104_2020_4975_MOESM3_ESM.jpg]

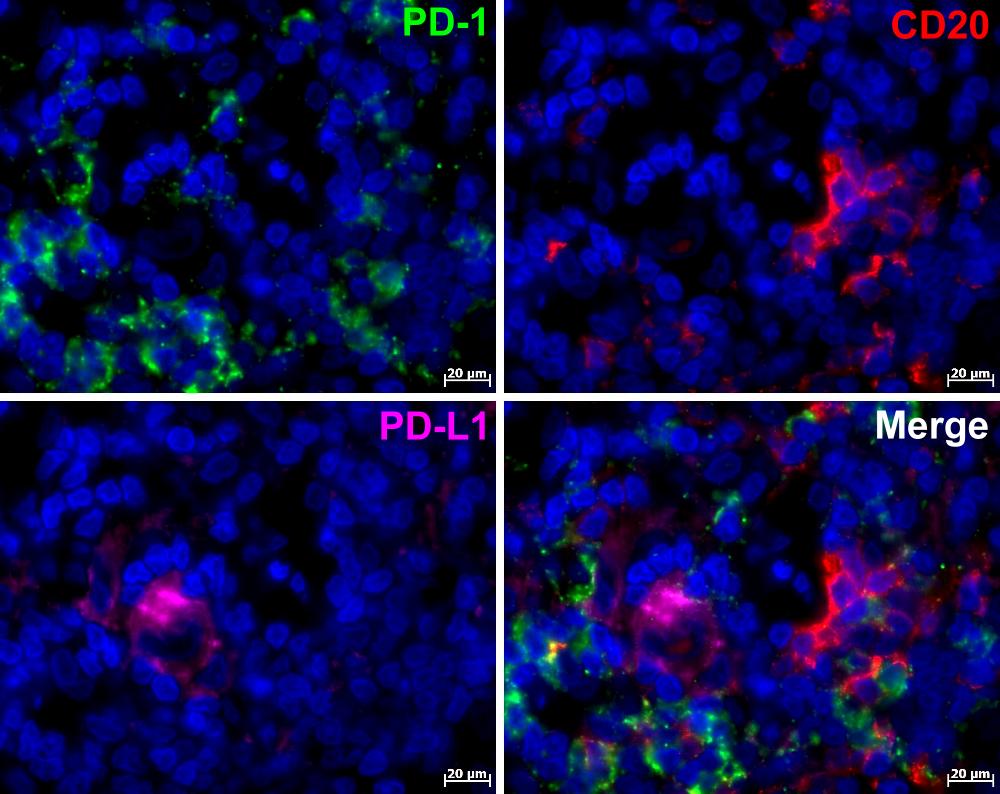

Supplement: Supplementary file 4 — Additional file 4: Figure S2. Immunofluorescent triple staining of PD-1, PD-L1 and CD20 in Hodgkin lymphoma: PD-1 (Alexa Fluor-488, green), PD-L1 (Alexa Fluor-647, magenta) and CD20 (Cy3, red). Nuclei counterstained with DAPI (blue channel). CD20 was not found in Reed/Sternberg cells (RSC). [file 13104_2020_4975_MOESM4_ESM.jpg]

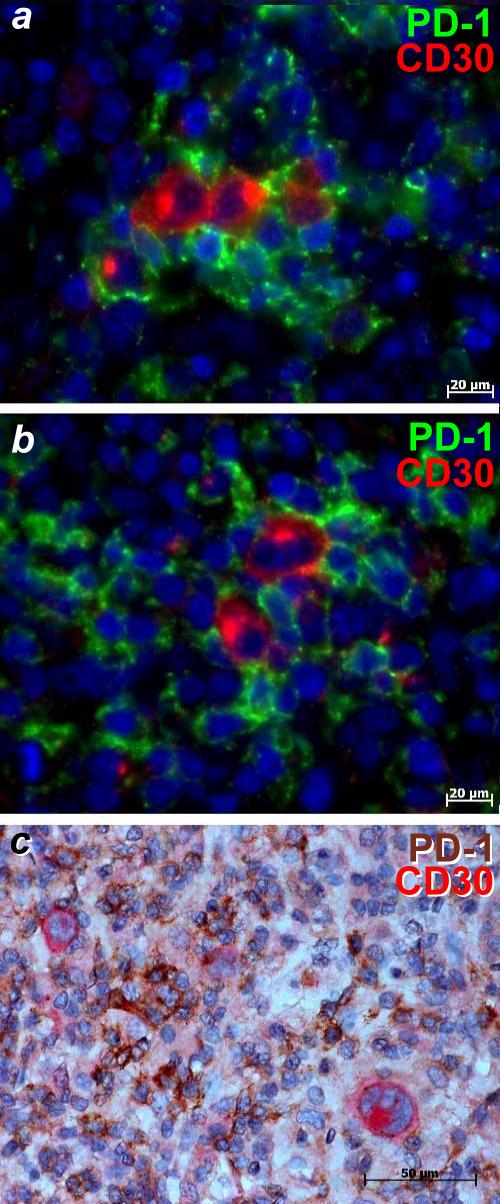

Supplement: Supplementary file 5 — Additional file 5: Figure S3. Double immunostaining of PD-1 and CD30 in Hodgkin lymphoma: (a, b) Immunofluorescent double staining of PD-1 (Alexa Fluor-488, green) and CD30 (Cy3, red). RSC intensely express CD30 in the cytoplasm and do not bear PD-1 antigen. (c) Double immunoenzyme staining for CD30 (red) and PD-1 (brown). Nuclei counterstained with hematoxylin. RSC do not bear PD-1 antigen. [file 13104_2020_4975_MOESM5_ESM.jpg]

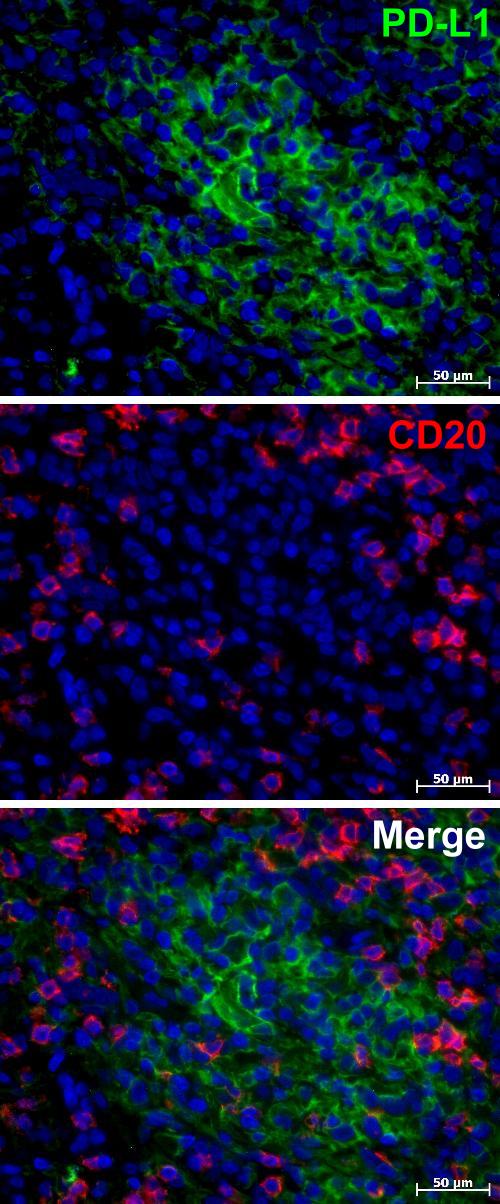

Supplement: Supplementary file 6 — Additional file 6: Figure S4. Immunofluorescent double staining of PD-L1 and CD20 in AITL: PD-L1 (Alexa Fluor-488, green), CD20 (Cy3, red). Nuclei counterstained with DAPI (blue channel). PD-L1+ cells do not co-express CD20. [file 13104_2020_4975_MOESM6_ESM.jpg]

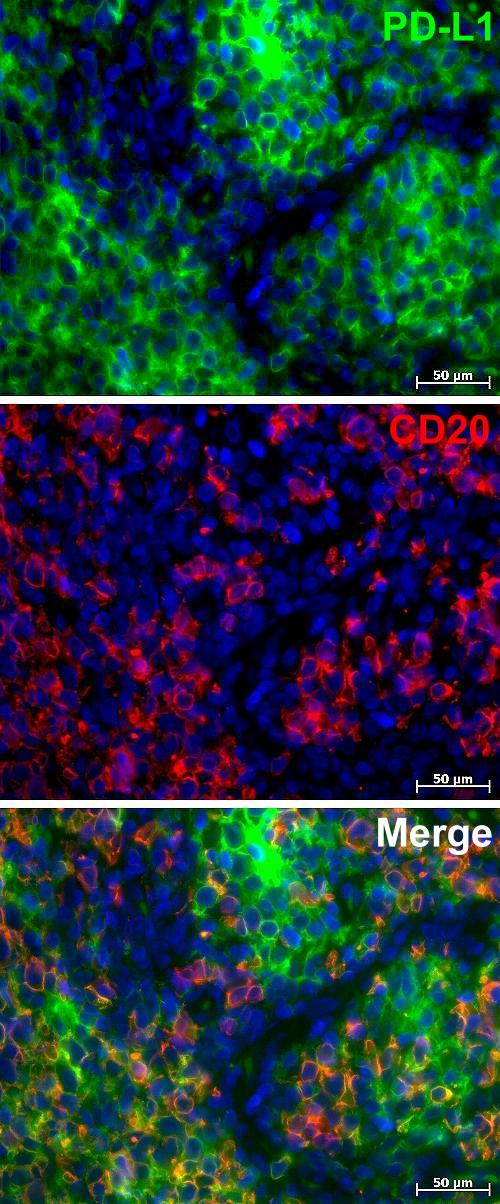

Supplement: Supplementary file 7 — Additional file 7: Figure S5. Immunofluorescent double staining of PD-L1 and CD20 in mediastinal lymphoma: PD-L1 (Alexa Fluor-488, green), CD20 (Cy3, red). Nuclei counterstained with DAPI (blue channel). Co-expression of PD-L1 and CD20 in the same cells in mediastinal lymphoma is manifested by a hybrid orange colour. [file 13104_2020_4975_MOESM7_ESM.jpg]

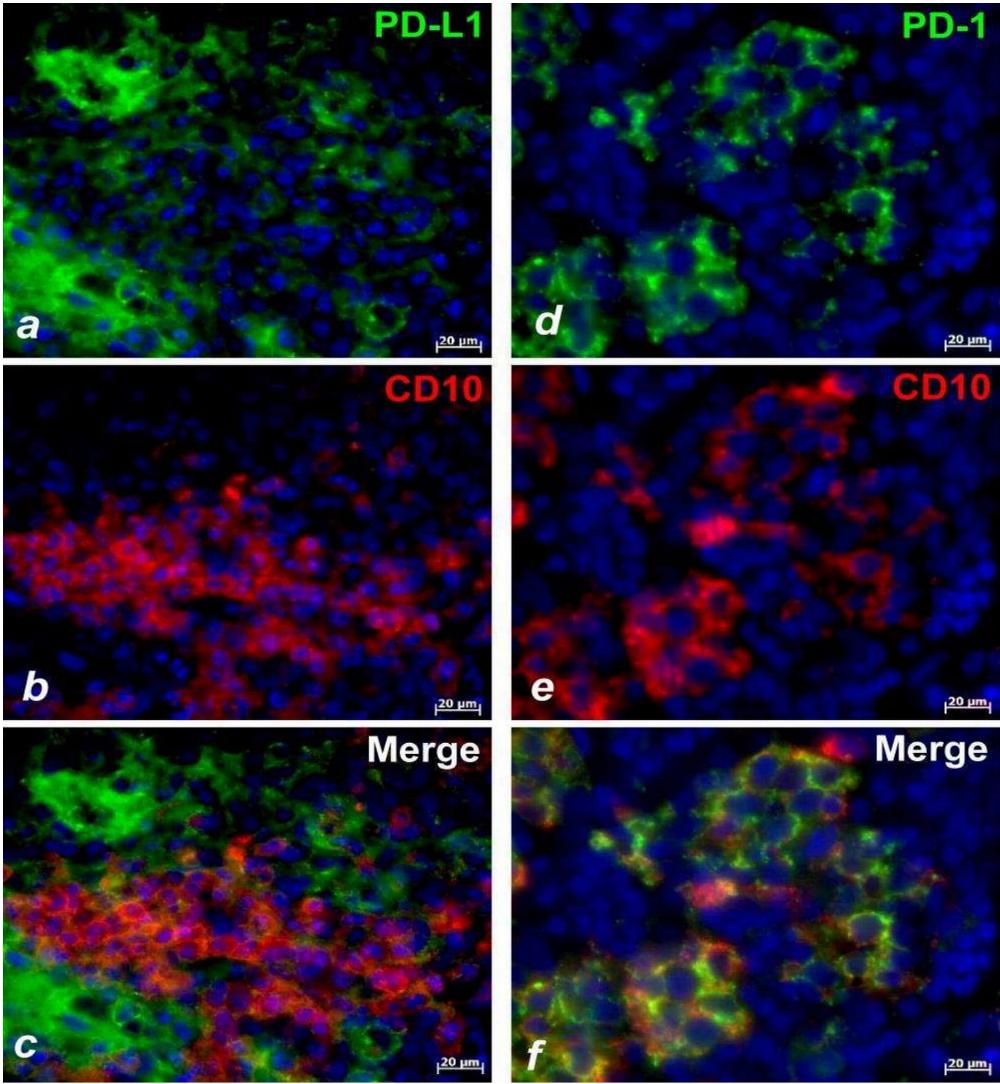

Supplement: Supplementary file 8 — Additional file 8: Figure S6. Immunofluorescent double staining of PD-L1 (FITC, green) and PD-1 (Alexa Fluor-488, green) vs CD10 (Cy3, red) in AITL. Nuclei counterstained with DAPI (blue channel). Whereas PD-L1 and CD10 were never found in the same cells (a–c), the majority of PD-1-positive cells revealed a co-expression of CD10 manifested by a hybrid orange coulour (d–f). [file 13104_2020_4975_MOESM8_ESM.jpg]
